# Data acquisition system for X-ray free-electron laser experiments at SACLA

**DOI:** 10.1107/S1600577515004506

**Published:** 2015-04-17

**Authors:** Yasumasa Joti, Takashi Kameshima, Mitsuhiro Yamaga, Takashi Sugimoto, Kensuke Okada, Toshinori Abe, Yukito Furukawa, Toru Ohata, Ryotaro Tanaka, Takaki Hatsui, Makina Yabashi

**Affiliations:** a Japan Synchrotron Radiation Research Institute, 1-1-1 Kouto, Sayo-cho, Sayo-gun, Hyogo 679-5198, Japan; b RIKEN SPring-8 Center, 1-1-1 Kouto, Sayo-cho, Sayo-gun, Hyogo 679-5148, Japan

**Keywords:** X-ray free-electron laser, shot-to-shot data acquisition, massive data analysis

## Abstract

A data acquisition system for X-ray free-electron laser experiments at SACLA has been developed. The system has been designed for reliable shot-to-shot data storage with a high data stream greater than 4 Gbps and massive data analysis. Configuration of the system and examples of prompt data analysis during experiments are presented. Upgrade plans for the system to extend flexibility are described.

## Abstract 2

A data acquisition system for X-ray free-electron laser experiments has been developed at SACLA. The system was designed for reliable shot-to-shot data storage with a high data stream greater than 4 Gbps and massive data analysis.

## 1. Introduction   

X-ray free-electron lasers (XFELs) with extreme brilliance and ultra-fast pulse duration (Emma *et al.*, 2010 ▸; Ishikawa *et al.*, 2012 ▸) provide us with new opportunities to observe complex targets with angstrom and femtosecond resolutions. In particular, a ‘diffraction before destruction’ concept (Neutze *et al.*, 2000 ▸) was proposed as a new experimental scheme using XFELs. Here, X-ray diffraction signals from samples are recorded within a femtosecond pulse duration while avoiding structural changes due to radiation damage, although samples are finally destroyed on a picosecond timescale. Since we must utilize fresh samples under different conditions for each XFEL pulse, shot-to-shot data acquisition (DAQ), to record and correlate the data that are associated with each XFEL pulse, is mandatory. We define ‘DAQ system’ as a system for data acquisition and analysis here. To exploit the full capabilities of an XFEL light source, the processing speed of the data acquisition must be compatible with the repetition rate of the XFEL source, typically several tens to one hundred Hz for that based on normal-conducting accelerators. In addition, shot-to-shot recording of the characteristics of XFEL pulses is necessary because of the stochastic nature of a self-amplified spontaneous emission (SASE) scheme which provides substantial fluctuations in beam properties. The effects on experimental data must be carefully analyzed. Based on these requirements, the XFEL DAQ system must be designed to provide fast handling capability for a massive data set. These requirements are very different from those for conventional synchrotron radiation sources, in which an integration-type X-ray detector is used and the data stream is much smaller.

As an example of massive data handling, we consider an experimental scheme based on serial femtosecond crystallography (SFX) (Chapman *et al.*, 2011 ▸; Boutet *et al.*, 2012 ▸), in which single-shot diffraction patterns from randomly oriented micro-crystals are recorded for every XFEL shot. At SACLA, we developed the DAPHNIS (diverse application platform for hard X-ray diffraction in SACLA) platform (Tono *et al.*, 2015 ▸; Sugahara *et al.*, 2014 ▸). For this platform, we employed a short-working-distance (SWD) multi-port charge-coupled device (MPCCD) octal-sensor detector, which has eight MPCCD sensor modules to measure diffraction patterns in a wide-angle region (Kameshima *et al.*, 2014 ▸). When we run an SFX experiment with a maximum repetition rate of 60 Hz at SACLA, we obtain 216000 diffraction patterns per hour, which corresponds to a high data throughput of 4 gigabits per second (Gbps), *i.e.* ∼1.6 terabytes (TB) per hour. As another example, we introduce coherent diffraction imaging (CDI) experiments, a lensless imaging technique using coherent X-rays combined with phase-retrieval algorithms (Fienup, 1982 ▸; Luke, 2005 ▸). We constructed a MAXIC (multiple application X-ray imaging chamber) platform (Song *et al.*, 2014 ▸) for CDI experiments at SACLA. To record diffraction signals in a wide dynamic range from small-angle to wide-angle regions, we combined two MPCCD detectors in a tandem arrangement, *i.e.* an MPCCD octal-sensor detector in the front and an MPCCD dual-sensor detector at the far end. The maximum data throughput (5 Gbps) for CDI experiments is slightly larger than that for SFX experiments.

For these experiments, all data from the detectors with the beam parameters should be reliably stored and properly analyzed in the DAQ system. For data storage, one needs a system that has a high-speed I/O performance required for fast data acquisition during experiments (*i.e.* high-speed storage, HSS), while a large capacity with a moderate speed is desirable for archiving the massive amount of data over the long term (*i.e.* large capacity storage, LCS). The moderate I/O performance is required to reduce the cost for LCS. For data analysis, we also need two different functions, as for the data storage. One is a prompt data analysis (PDA) function to optimize the experimental conditions during experiments, as well as to reduce the amount of targeting data, which is achieved by applying a data screening process. The term ‘PDA’ is used to mean both analyses of the ‘live’ data before storage and of the recorded data. The other is a full data analysis (FDA) function to perform comprehensive investigations after the experiments are completed.

In this report, we describe a DAQ system for XFEL experiments at SACLA. The system was designed to achieve HSS, LCS, PDA and FDA functions with high efficiency. In §2, we describe the system configuration; §3 provides detailed examples of the PDA; finally, §4 describes future perspectives for the system.

## 2. Configuration of the DAQ system   

**Figure 1:**
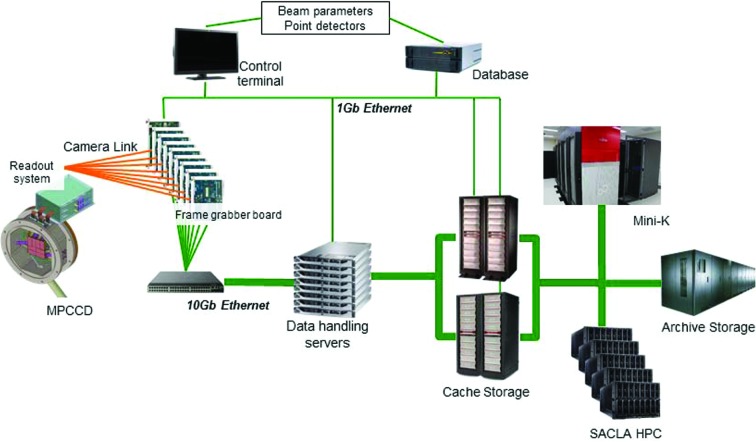
Schematic configuration of the DAQ system at SACLA. The system consists of a front-end system (VME-based frame-grabber boards), data-handling servers, database, cache storage system, SACLA HPC system, archive storage system and mini-K system. Two independent cache storage systems, one for BL2 and the other for BL3, are in operation.

Fig. 1 ▸ shows the schematic configuration for the DAQ system at SACLA. The timing signal of the master-trigger system to drive the accelerator equipment at 60 Hz cycle is delivered to the DAQ system so that the detectors could be operated exactly in synchronization with the timing of the XFEL pulse (Yamaga *et al.*, 2011 ▸). The signals from the detectors are preprocessed in a front-end system and sent to data-handling servers and a cache storage system working as an HSS system. The DAQ system could include other large non-imaging data devices (*e.g.* fast-sampling ADC waveforms) in addition to MPCCD detectors by installing data-handling servers dedicated to the data transfer. The PDA function is achieved by the data-handling servers and a SACLA high-performance computer (HPC), which receives the data from the cache storage system. After a designated grace period (typically six months), to ensure enough disk space for HSS, the data in the cache system are transferred to an archive storage system serving as an LCS system. For the FDA function, we utilize a mini-K supercomputer system, which is compatible with the K-computer, a 10-PFLOPS supercomputer developed by RIKEN AICS (http://www.aics.riken.jp/en/). We constructed a relational database (DB) for recording the beam parameters and signals from point detectors such as photodiodes (Tono *et al.*, 2013 ▸). Linking the data in these systems to a specific XFEL shot is achieved by using tag information that is added to each data point. The ‘tag’ embedded for each data ensures that the various pieces of data are correctly associated with the correct shot. We define a ‘run’ as an experimental unit to bundle the consecutive shot-to-shot data. We constructed *RunControlGUI*, which is a control software featuring a graphical user interface (GUI), for conducting experiments using a control terminal. Backbone software and the GUI for the DAQ system at SACLA were developed using C/C++ and Python, respectively.

Below, we explain in more detail the function of each component in the DAQ system. Note that specifications for the computer systems are summarized in Table 1 ▸.

### 2.1. Front-end system   

The front-end system converts signals of the MPCCD detectors from Camera Link protocol to Ethernet protocol by using a VME-based frame-grabber board. The total number of boards is 12. It then sends these data to the data-handling servers *via* TCP/IP.

### 2.2. Data-handling servers   

The purpose of the data-handling servers as an HSS system is to buffer the data before sending it to the cache storage system. The total number of the data-handling servers is 16. We enable parallel data transfer by using a set of data-handling servers, achieving throughput greater than 4 Gbps. The data-handling servers work well for simple PDA processing, such as calculation of average diffraction intensities at a region of interest (ROI). These results are recorded in the DB (see §3.1).

### 2.3. Cache storage system   

The cache storage system functions as an HSS system. The data from eight (or two) panels of the MPCCD octal (or dual) sensor are stored separately in the storage system. Two independent cache storage systems, one for BL2 and the other for BL3, are in operation. BL2 and BL3 are the hard X-ray beamlines of SACLA. Both storage systems are capable of I/O throughput greater than 4 Gbps.

### 2.4. Archive storage system   

The archive storage system serving as an LCS system is a hierarchical storage system consisting of hard disk drives and tape drives. The archive system is used for the raw detector data as well as for the user’s data after PDA and FDA.

### 2.5. Database (DB)   

The MySQL DB is used for recording beam parameters and signals as well as for managing data for experiments using information on the ‘run’ (see §3.2). For load distribution and backup, the data in the DB are replicated to a few servers, while the data stream of the replication is small enough (<5 Mbps) not to interfere with the total data traffic.

### 2.6. SACLA HPC system   

We designed the SACLA HPC system to perform PDA efficiently. The system consists of 81 computer nodes, a local fast storage device, and a login node. For fast data transfer, all computer nodes in the SACLA HPC system are connected to the cache storage systems with a fast file system (StorNext FS/GPFS) *via* 10 Gb Ethernet. The computer nodes are also connected to the mini-K system and to the archive storage system.

### 2.7. Mini-K supercomputer system   

The theoretical peak performance of the mini-K is about 1% of that of the K-computer. Since computer nodes in the system are connected *via* a highly scalable and reliable interconnect (Tofu 6-dimensional Mesh/Torus architecture), we can efficiently perform FDA computations such as correlation analysis between diffraction patterns.

## 3. Examples of PDA: data screening, data assembly and image visualization   

In this section we introduce some examples of PDA using the DAQ system: data screening, data assembly and image visualization.

### 3.1. Data screening using a ‘low-level filter’   

A set of X-ray images measured in XFEL experiments, such as SFX and CDI, can include extraneous data. One of the main reasons is due to a ‘failed shot’, where an XFEL pulse does not hit a sample at all. We have developed a *LowLevelFilter* (*LLF*) to screen these data. In the *LLF*, maximum or average intensities of an ROI calculated in the data-handling servers are recorded shot-to-shot in the DB. The values are used to select targeting data (‘hit’ data) that satisfy specific criteria set by users. Using the *LLF*, we can reduce the number of diffraction patterns transferred to the SACLA HPC and thus reduce the time required for PDA (see also §3.2).

**Figure 2:**
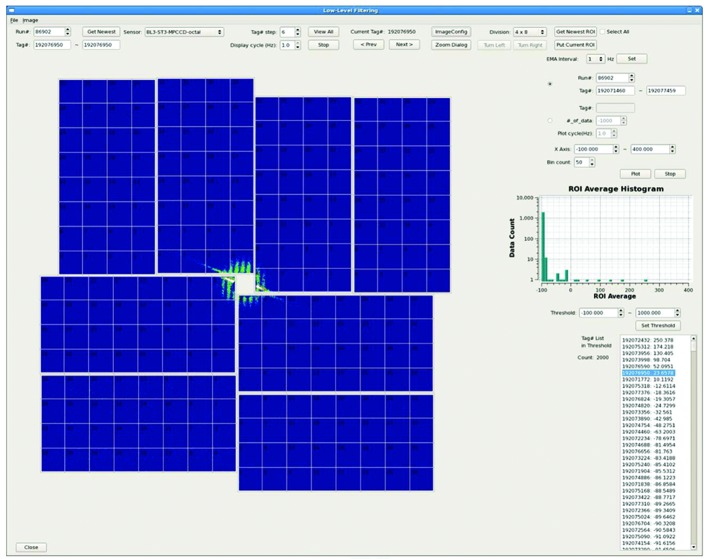
Screen image of the *LowLevelFilter* (*LLF*) GUI. An assembled image of the MPCCD octal-sensor detector is shown on the left. Using this panel, users can select one or multiple regions as ROI(s). A histogram of the maximum or the average intensities of the ROI is displayed in the upper right-hand corner. A list of tag numbers with the ROI values in descending order is displayed in the lower right-hand corner. The number of targeting data that satisfy the user criteria is also displayed on the left-hand side of the tag number list.

Fig. 2 ▸ shows a screen image of the GUI for *LLF*. Each MPCCD sensor image is divided into 4 × 8 or 8 × 16 square regions. Users can select one or multiple regions as ROI(s). A histogram of the maximum or the average intensities of the ROI, and a list of tag numbers with the ROI values in descending order, are displayed in the *LLF* GUI to help users to set a threshold that discriminates a ‘hit’ condition. To evaluate the hit rate during experiments, the number of targeting data that satisfy the user criteria is also displayed in the GUI. In the case of typical SFX experiments, we can eliminate about two-thirds of the total images using *LLF* (the fraction depends on the sample condition).

### 3.2. Data assembly with *DataConvert*   

We need to combine experimental data located in various parts of the cache storage systems and the DB into a single file for data portability. For this purpose we developed *DataConvert* software that is executed at the computer nodes in the SACLA HPC. Using information on a ‘run’ in the DB, *DataConvert* combines the raw detector data with the DB data, and converts it into a ‘SACLA Run Data Format’ which is based on the HDF5 file format (Hierarchical Data Format, http://www.hdfgroup.org/HDF5). HDF5 is supported by many software platforms, including C/C++, Fortran, Python, Java and MATLAB. The conversion carries out the calibration of the detector sensor (Kameshima *et al.*, 2014 ▸), except for background (dark frame) subtraction. *DataConvert* can reconstruct an assembled image from the eight (or two) panels of the MPCCD octal (or dual) sensor. In ‘reconstruction’ mode, gain calibration among the sensor panels is automatically performed.

The speed of single-stream *DataConvert* is ∼2 Hz to gather data with the MPCCD octal sensor detector (∼20 Hz per single-sensor). To enable efficient data analysis, *DataConvert* can assemble only the data in a hit condition that has been screened by the *LLF* process, as shown in §3.1. Users can also select a specific data set by designating a list of tag numbers, which are connected to the beam status recorded in the DB.

The *DataConvert* processing works after an experimental ‘run’ is finished. When the processing time of a PDA is comparable with that for the ‘run’, the PDA to monitor experimental conditions could be performed in parallel with experiments, although there is a time lag. For example, data assembly with the *LLF* and indexing by *CrystFEL* (White *et al.*, 2012 ▸) are conducted using 16 computer nodes, which enables monitoring of the hit rate and the indexing rate during SFX experiments. Here a set of the experimental data for a ‘run’ is decomposed into subsets. Each computer node executes *DataConvert* and subsequent *indexamajig* of *CrystFEL* for a subset independently, and then a master computer node summarizes the results.

The latest version of *DataConvert* has been developed using a *DataAccessAPI* that is a set of separate functions for pulling the data stored in the cache storage systems and the DB. *DataAccessAPI* will be available to users so that user programs could directly gather experimental data needed for their analysis.

### 3.3. Image visualization   

**Figure 3:**
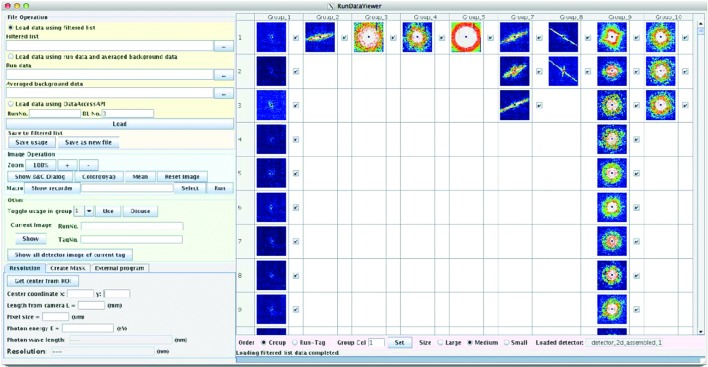
Screen image of *RunDataViewer*. An example of the classification of diffraction patterns is shown. Each column corresponds to a group of the classification.

In CDI experiments, image visualization is essential for evaluating the quality of the experimental data. Tools to facilitate the visualization of a large amount of X-ray images are useful for increasing the efficiency of the evaluation. Experimental data sets often contain unwanted contamination, *e.g.* diffraction signal from unwanted substances. Classification of the diffraction patterns (Yoon *et al.*, 2011 ▸) is useful for identifying such data. We developed *DataSort* software for classifying diffraction patterns by using characteristic values, which can be selected from, for example, a set of average intensities of ROIs or the radial average of the pattern. These characteristic values from each snapshot image are represented as a vector in a multidimensional space. The resulting set of vectors is divided into groups using a *k*-means algorithm (MacQueen, 1967 ▸) or a Gaussian mixture model (GMM) (McLachlan & Basford, 1988 ▸). The processing speed is much faster than those for the data acquisition and for the data assembly. The images after the classification can be visualized using a *RunDataViewer*, which was developed as a plug-in for *ImageJ* (Schneider *et al.*, 2012 ▸). *DataSort* and *RunDataViewer* are executed at the computer nodes in the SACLA HPC. Fig. 3 ▸ shows an example of the classification of diffraction patterns.

## 4. Future perspectives for the DAQ system   

In this section we describe upgrade plans for the DAQ system at SACLA to extend user flexibility.

So far, most of the PDA processes are performed for the recorded data in the cache storage system, although this scheme requires a time lag for analysis because in this case users cannot operate the PDA processes before an experimental ‘run’ is finished. The delay is several minutes for typical SFX experiments. To reduce this delay, we are developing an online analysis system with an *OnlineAPI* as a user application programming interface (API), so that user programs such as *CASS* (Foucar *et al.*, 2012 ▸) and *Cheetah* (Barty *et al.*, 2014 ▸) can access the ‘live’ data in the data-handling servers before it reaches the cache storage system, similar to an implementation developed for the *LLF*.

We are also developing a software framework so that experimental users, who may not be familiar with parallel computing, can easily and effectively perform PDA and FDA. The framework decouples user programs that are executed at a computer node from parallelization among computer nodes. Such a framework should contribute to the acceleration of XFEL scientific achievements.

Finally, reliable shot-to-shot data storage and massive data analysis developed for the XFEL DAQ system are greatly needed for the full utilization of next-generation synchrotron radiation sources, which require a DAQ system with a much higher data stream using advanced high-speed two-dimensional detectors that operate at an output data bandwidth of around 20 Gbps per single sensor (see, for example, http://rsc.riken.jp/pdf/SPring-8-II.pdf). The concepts discussed in this report can be extended to next-generation DAQ systems, which should have HSS, LCS, PDA and FDA functions to satisfy the requirement for high-speed data acquisition and massive data analysis.

## Figures and Tables

**Table 1:** Specifications for large computer systems at SACLA

	Specifications	
Cache storage system	System for BL2 experiments	
	Hardware:	DDN S2A9900
	Operating system for servers:	RHEL 5.4
	Storage capacity:	∼200 TB
	File system:	StorNext FS (I/O ∼5 GB s^−1^ > 4 Gbps)

	System for BL3 experiments	
	Hardware:	DDN SFA10K
	Operating system for servers:	RHEL 5.8
	Storage capacity:	∼250 TB
	File system:	GPFS (I/O ∼5 GB s^−1^ > 4 Gbps)

Archive storage system	Hardware:	Disk: DDN SFA10K
		Tape: IBM System Storage TS3500 and TS1140 (five drives)
	Operating system for servers:	RHEL 5.8
	Storage capacity:	Disk: 1 PB; tape: 7 PB (can be increased up to 28 PB)
	File system of the disk part:	GPFS (I/O > 4 GB s^−1^)
	Hierarchical management:	Tivoli Storage Manager (I/O ∼200 MB s^−1^)

HPC system	Hardware:	DELL PowerEdge M1000e and M610 (80 nodes), R910 (1 node),
		Storage: DDN SFA10K
	Operating system for nodes:	CentOS 5.6 Theoretical peak performance: ∼13 TFLOPS ([intel X5690 × 2] × 80 + [Intel X7650 × 4] ×1)
	Total memory capacity:	∼3 TB (24 GB × 80 + 1 TB × 1)
	Interconnect:	Infiniband QDR
	Storage capacity:	∼170 TB
	File system:	Lustre (I/O ∼7 GB s^−1^)

Mini-K system	Supercomputer	
	Hardware:	Fujitsu PRIMEHPC FX10 4 rack model
	Operating system for nodes:	Linux-base OS for FX10 (by Fujitsu)
	Theoretical peak performance:	∼90 TFLOPS (SPARC64 IXfx × 384)
	Total memory capacity:	∼12 TB (32 GB × 384)
	Interconnect:	Tofu 6D Mesh/Torus architecture
	Storage capacity:	∼600 TB (global: 500 TB, local: 100 TB)
	File system:	FEFS (I/O global: ∼5 GB s^−1^, local: ∼10 GB s^−1^)
	
	Storage for efficient data transfer	
	Hardware:	DDN SFA12K
	Storage capacity:	∼1 PB
	File system:	Lustre (I/O ∼10 GB s^−1^)
